# RhoA/ROCK1 regulates Avian Reovirus S1133-induced switch from autophagy to apoptosis

**DOI:** 10.1186/s12917-015-0417-6

**Published:** 2015-05-06

**Authors:** Ping-Yuan Lin, Ching-Dong Chang, Yo-Chia Chen, Wen-Ling Shih

**Affiliations:** Department of Biological Science and Technology, Pingtung, 91201 Taiwan; Veterinary Medicine, National Pingtung University of Science and Technology, Pingtung, Taiwan; Graduate Institute of Biotechnology, National Pingtung University of Science and Technology, 1, Shuefu Rd., Neipu, Pingtung, 91201 Taiwan

**Keywords:** ARV S1133, Autophagy, Apoptosis, RhoA, ROCK1

## Abstract

**Background:**

Autophagy is an essential process in the control of cellular homeostasis. It enables cells under certain stress conditions to survive by removing toxic cellular components, and may protect cells from apoptosis. In the present study, the signaling pathways involved in ARV S1133 regulated switch from autophagy to apoptosis were investigated.

**Results:**

ARV S1133 infection caused autophagy in the early to middle infectious stages in Vero and DF1 cells, and apoptosis in the middle to late stages. Conversion of the autophagy marker LC3-I to LC3-II occurred earlier than cleavage of the apoptotic marker caspase-3. ARV S1133 also activated the Beclin-1 promoter in the early to middle stages of infection. Levels of RhoA-GTP and ROCK1 activity were elevated upon ARV S1133 infection, while inhibition of RhoA and ROCK1 reduced autophagy and subsequent apoptosis. Conversely, inhibition of caspase-3 did not affect the level of autophagy. Beclin-1 knockdown and treatment with autophagy inhibitors, 3-MA and Bafilomycin A1, suppressed ARV S1133-induced autophagy and apoptosis simultaneously, suggesting the shift from autophagy to apoptosis. A co-immunoprecipitation assay demonstrated that the formation of a RhoA, ROCK1 and Beclin-1 complex coincided with the induction of autophagy.

**Conclusion:**

Our results demonstrate that RhoA/ROCK1 signaling play critical roles in the transition of cell activity from autophagy to apoptosis in ARV S1133-infected cells.

## Background

Avian reoviruses (ARVs) are members of the genus Orthoreovirus, which belongs to the Reoviridae family [1]. ARVs are non-enveloped viruses that contain 10 double-stranded RNA genomic segments and several encoded proteins, including at least 10 structural proteins and 4 nonstructural proteins [2,3]. ARVs cause many poultry diseases, including malabsorption syndrome, chronic respiratory disease and arthritis. In contrast to mammalian reoviruses, ARVs cause massive fusion of host cells but are deficient in hemagglutination activity [4]. The pathogenesis of ARV-induced apoptosis has been studied in ARV-infected chicken tissues, including the intestine, tendon, liver, and bursa [5]. We previously reported that ARV S1133 induces apoptosis by modulating Src, p53, mitogen-activated protein kinase (MAPK), and protein kinase C δ, and also elicits cytochrome c release from mitochondria to the cytosol [6-8]. ARV S1133 encodes nonstructural protein p10, which mediates cell syncytium formation through the activation of the small GTPase, RhoA, and Rac1 signaling [9]. Autophagy is a basic bulk degradation mechanism that controls the recycling and clearance of intracellular constituents into double-membrane vesicles, and traffic of these vesicles to the lysosomes for continued cell survival [10]. Formation of autophagosomes requires over 15 autophagy-related proteins, including microtubule associated protein 1 light-chain 3, the UNC53-like kinase1 (ULK1) complex, and PI3Ks [11]. Autophagy is tightly regulated by several cellular signaling pathways, and the major negative regulator is the serine/threonine kinase mammalian target of rapamycin (mTOR); however, the class III PI3K/Beclin-1 pathway positively regulates autophagy [12]. Autophagy has been recognized as a stress response to enable eukaryotic organisms to survive during severe conditions, such as nutrient starvation, oxidative stress, chemicals and infection by intracellular pathogens [13,14].

Previous studies on intestinal epithelial cells, demonstrated that oncogenic Ras increases RhoA activity and promotes the degradation of Beclin-1, leading to the subsequent inhibition of autophagy [15].Under nutrient deprivation conditions, the activation of ROCK1 (Rho-associated, coiled-coil containing protein kinase 1) promotes autophagy by binding to and phosphorylating Beclin-1 at Threonine 119. This threonine phosphorylation inhibits the ROCK1 activity and promotes the association of Beclin-1 with Bcl-2. Thus, ROCK1 is one of the upstream regulators of Beclin-1-mediated autophagy and controls the homeostasis between autophagy and apoptosis [16]. ARV S1133 induces autophagy in both primary chicken embryonic fibroblast cells (CEF) and African green monkey kidney cells (Vero), and infection with this virus increases the number of double-membrane vesicles in these cells through the PI3K/Akt/mTOR pathway [17]. ARV nonstructural protein p17 is an activator of autophagy and regulates the expression of Beclin-1 and LC3 [18]. Thus, ARV S1133 seems to modulate autophagy and apoptosis *via* specific host cell signaling mechanisms. We hypothesized the existence of a switch between the kinetic control of these two kinds of programmed cell death during the ARV S1133 replication cycle. Autophagic cell death could occur in condition which without the involvement of apoptosis or necrosis [19]. Additionally, apoptosis and autophagy can simultaneously occur or exert synergistic effects under the same stress conditions, whereas in certain situations autophagy triggered only when apoptosis is inhibited [20,21]. Some studies have linked these two different types of programmed cell death; however, there exist intricate relationships between them, the significance and precise regulation are controversial [22]. In this study, we investigated the cross-talk between autophagy and apoptosis in ARV S1133-infected cells. We aimed to determine whether a molecular association exists between autophagy and apoptosis, and to elucidate the relationship between these cell death modes.

## Results

### Kinetics of autophagy and apoptosis in ARV S1133-infected DF1 and Vero cells

**Figure 1:**
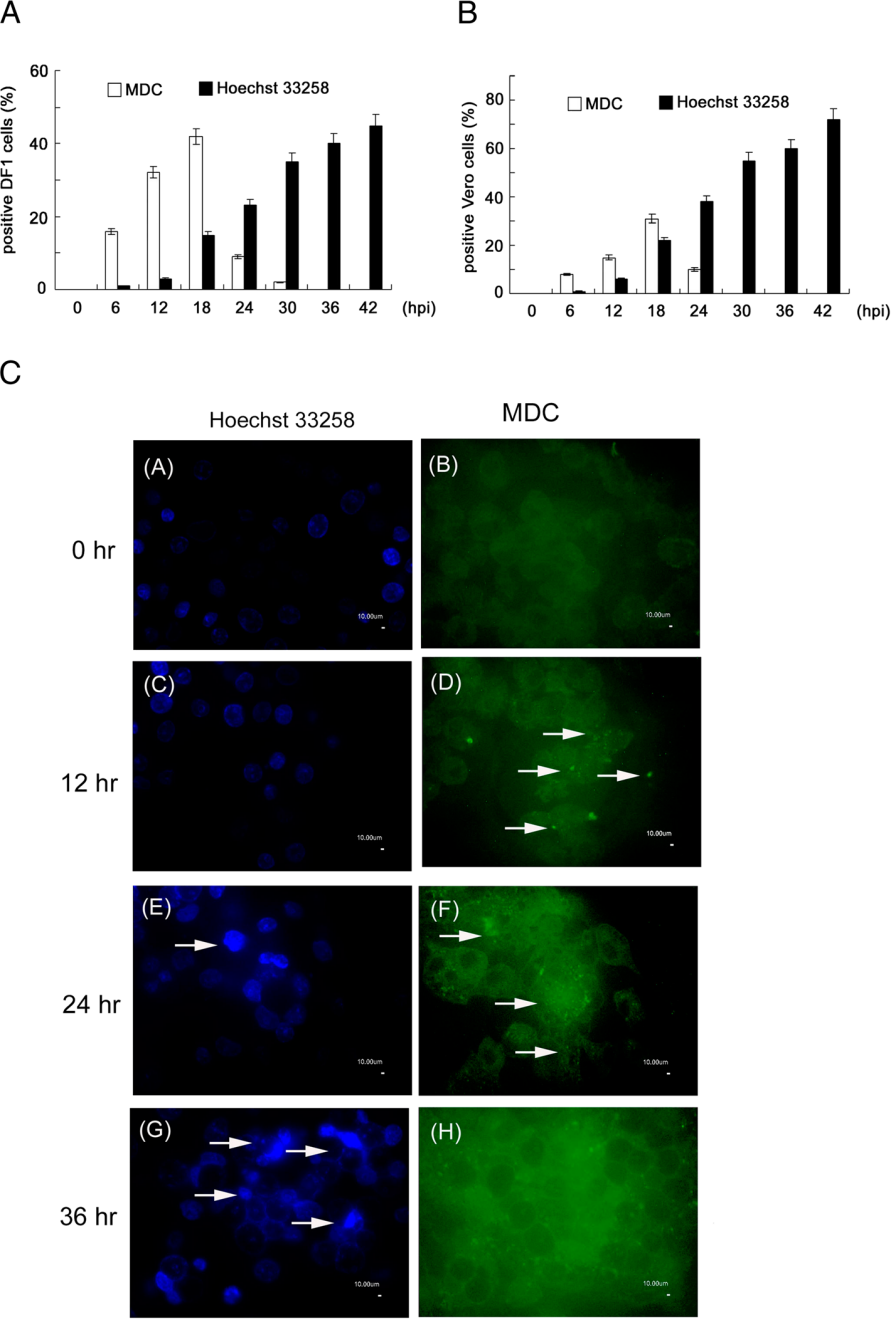
ARV S1133 induces autophagy and subsequent apoptosis in cultured cells. **(A)** DF1 cells infected with ARV S1133 at an MOI of 20. **(B)** Vero cells infected with ARV S1133 at an MOI of 5 for 0–42 hr. At the indicated time points, cells were stained with monodansylcadaverine (MDC) or Hoechst 332588. The percentage of positive cells was calculated for 20 independent fields at a magnification of 200×. **(C)** Vero cells infected with ARV S1133 at an MOI of 5 for 0–36 hr. In Hoechst 33258-stained cells (bright blue), arrows indicate apoptotic nuclei with condensed chromatin. In MDC stained cells, arrows indicate the autophagic vacuoles (×400 magnification, scale bar 10 μm).

**Figure 2:**
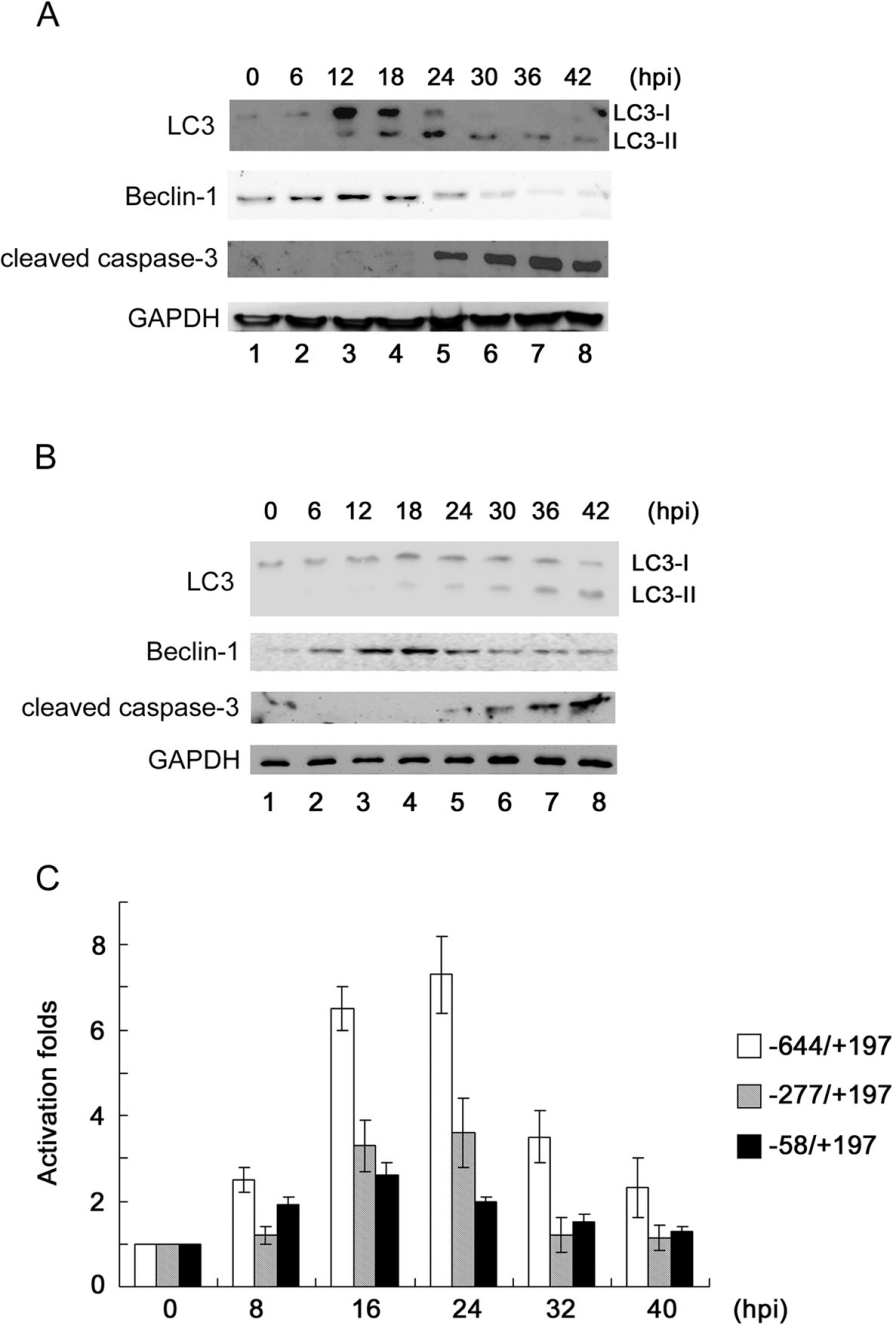
Upregulation of autophagic and apoptotic effectors and the Beclin-1 promoter by ARV S1133. **(A)** Vero cells infected with ARV S1133 at an MOI of 5 **(B)** DF1 cells infected with ARV S1133 at an MOI of 20. At the indicated time points, total cell lysates were collected. 30 μg of the extracted protein was separated by SDS-PAGE and transferred to a PVDF membrane. The expression of specific proteins was detected using the indicated antibodies. **(C)** Luciferase assay. Vero cells were transfected with three luciferase reporters with different lengths of the 5′-end regulatory region of the Beclin-1 gene. Following incubation with ARV S1133 for different time periods, the luciferase and β-galatosidase activities were measured and normalized to the transfection efficiency. The promoter activation levels were calculated against time zero. All experiments were performed three times, each in duplicate. The data are presented as the mean ± standard deviation (SD).

To identify the kinetic differences between autophagy and apoptosis, the autophagic and apoptotic cell percentages were first examined simultaneously in ARV S1133-infected cultured cells. The percentages of MDC- and Hoechst 33258-positive DF1 cells infected with ARV S1133 were evaluated by direct counting. Figure 1A shows the changes in the level of cell death during 42 hr of incubation. Autophagic cell death appeared at 6 hpi, increased at 12–18 hpi, decreased at 24 hpi, and disappeared at 30 hpi. However, a large number of apoptotic cells emerged at 18 hpi and continued to accumulate until the end of the observation period. A similar cell death trend was observed in ARV S1133-infected Vero cells (Figure 1B). At the molecular level, we analyzed the expression of microtubule-associated protein1 light chain 3 (LC3) and caspase-3. LC3-I conversion to LC3-II is a reliable marker of autophagosome formation [23,24], and caspase-3 cleavage is a well-established apoptotic index. The fluorescent staining shown in Figure 1C indicates the presence of autophagosomes and apoptotic nuclei. Significant numbers of MDC-labeled fluorescent particles accumulated between 12 hpi and 24 hpi; however this level decreased at 36 hpi. Apoptotic cells with condensed DNA appeared at the middle to late stages of ARV S1133 infection; from 24 hpi to 36 hpi. Figure 2A and B show that LC3 conversion and induced expression of Beclin-1 occurred in the early to middle infectious stages then disappeared gradually in both Vero and DF1 cells; whereas cleaved caspase-3 appeared in the middle of the infectious stage and continued to accumulate in the late stage.

In addition, we investigated whether ARV S1133 affects the expression of Beclin-1, a protein that participates in the regulation of autophagy, by analyzing the transcription of the 5′-flanking regions, from nucleotides −644 to +197, −277 to +197, and −58 to +197, of the *Beclin-1* gene. Our results demonstrated that these 5′-flanking regions constructed with a luciferase reporter were regulated by ARV S1133. Upon ARV S1133 infection, the −644 to +197 regulatory region of the Beclin-1 gene showed a higher luciferase activity than the −277 to +197 control region reporter, and the −58 to +197 region revealed a negligible luciferase response (Figure 2C).

### Activation of RhoA signaling by ARV S1133

**Figure 3:**
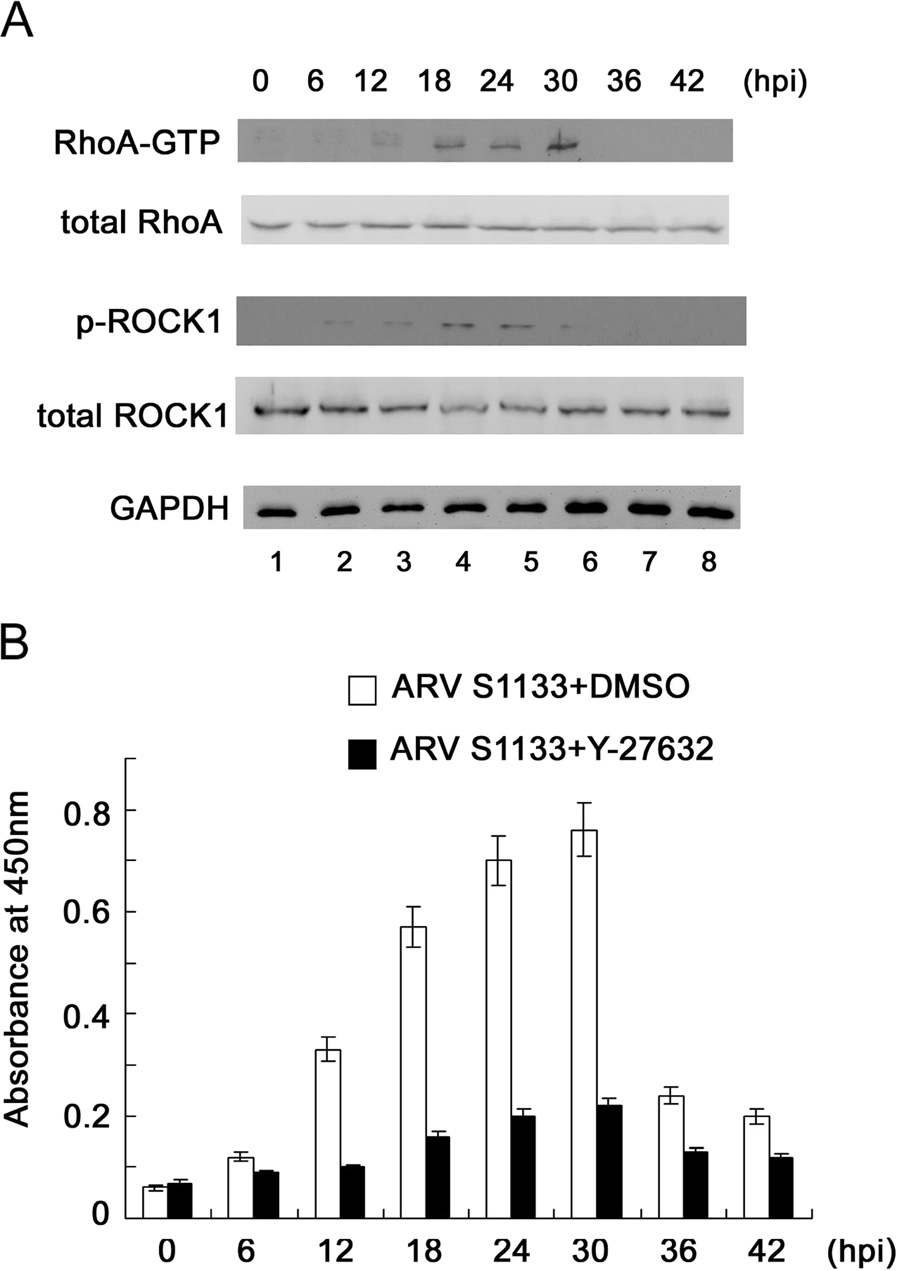
ARV S1133 activates RhoA and ROCK1.Vero cells infected with ARV S1133 at an MOI of 5 for different time periods. **(A)** Cell lysates reacted with GST-tagged Rhotekin Rho binding domain fusion protein. Active GTP-bound Rho was eluted and subsequent detection was performed by immunoblot analysis using anti-RhoA. Equal amounts of total cell lysates were subjected to SDS-PAGE and the protein levels were detected using the indicated antibodies. **(B)** 200 μg total cell lysates were added to the plates and incubated, and a detection antibody and substrate were then applied. The amount of phosphorylated substrate was measured at an absorbance of 450 nm. Each experiment was performed three times, each in duplicate. The data are presented as the mean ± SD.

We next investigated whether the autophagic pathway is regulated by small GTPase by analyzing the possible roles of RhoA signaling in ARV S1133-mediated autophagy and apoptosis. Our results showed that the levels of GTP-bound RhoA and phosphorylated ROCK1, a downstream effector of RhoA, were significantly increased 12 hpi, and this continued until the end of the middle stage (Figure 3A, lanes 3–6). To confirm the activation of ROCK1, the ELISA method was used to quantify the level of active ROCK. The results shown in Figure 3B indicate that the ARV S1133-elicited ROCK activity increased between 12 and 30 hpi. Additionally, the activity of ROCK was fully suppressed in the presence of ROCK inhibitor Y-27632, which further confirmed that the RhoA/ROCK pathway was involved and was active as ARV S1133-infected cells were undergoing autophagy.

### RhoA and ROCK1 are required for ARV S1133-induced autophagy and subsequent apoptosis

**Figure 4:**
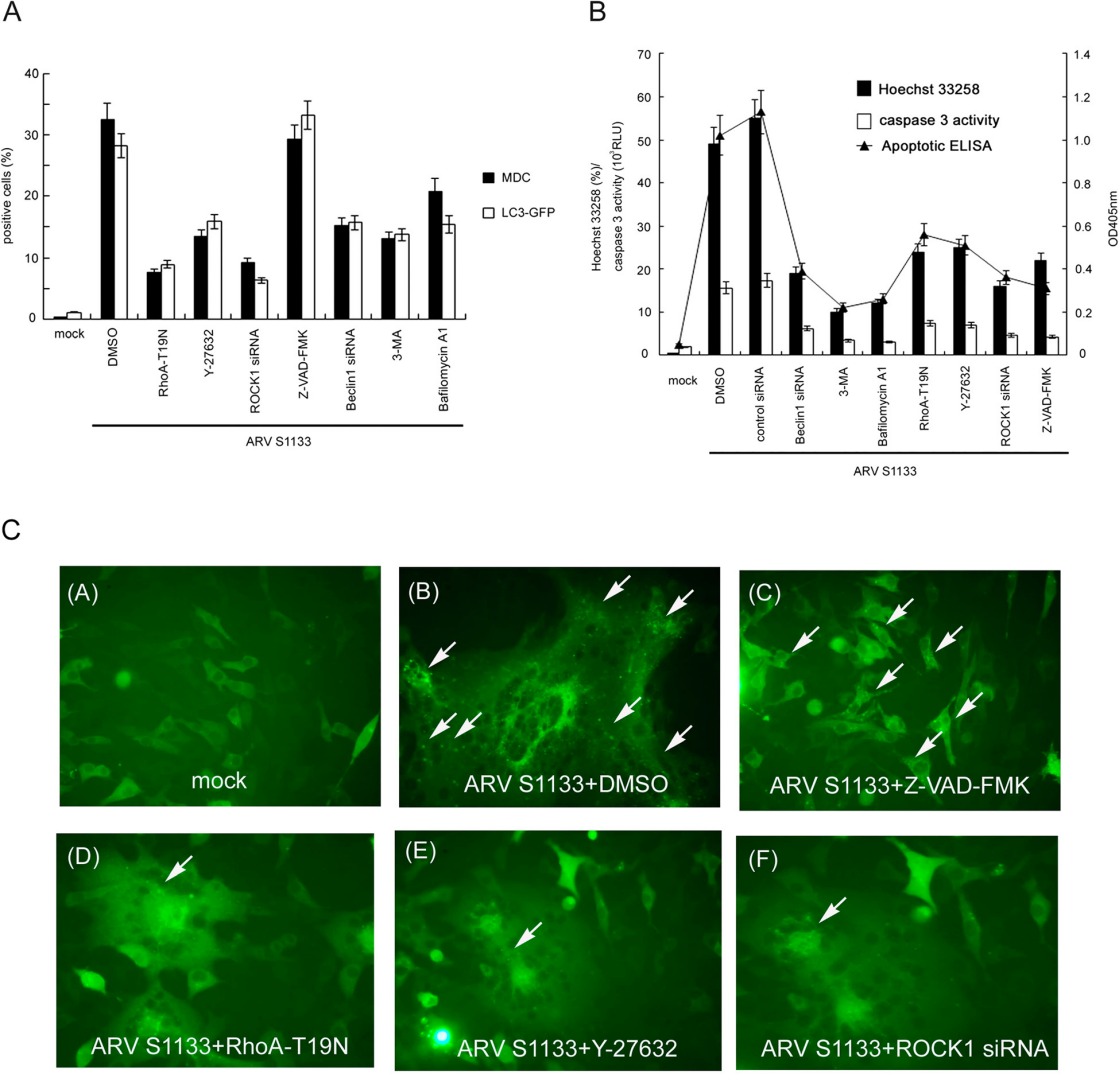
RhoA and ROCK1 are essential for ARV S1133-induced autophagy, apoptosis, and the conversion of autophagy to apoptosis. **(A)** Vero cells transfected with RhoA-T19N and incubated for 24 hr, before being transfected with siRNA for 72 hr, pre-treated with 5 μM Y-27632, 10 μM Z-VAD-FMK, 50 μM 3-MA and 0.1 μM Bafilomycin A1 at non-toxic concentrations for 4 hr, and then infected with ARV S1133 at an MOI of 5 for an additional 18-hr incubation. Autophagic vacuoles were stained with MDC, and the percentage of positive cells was calculated in 20 independent fields at a magnification of 200× (black bar). Vero cells transfected with LC3-GFP, together with RhoA-T19N, and incubated for 24 hr, or transfected with siRNA for 48 hr then transfected with LC3-GFP, or pretreated with inhibitor for 4 hr then transfected with LC3-GFP. 24 hr after LC3-GFP transfection, ARV S1133 was added for an additional 18-hr incubation period. GFP-positive cells containing more than 3 dots were counted as positive LC3-GFP cells. The percentage of positive cells was calculated in 20 independent fields at a magnification of 200× (white bar). **(B)** ARV S1133 infected cells at an MOI of 5 with an incubation period of 36 hr with identical treatment timings to those described in **(A)**. Three apoptosis assays were performed. The left y-axis represents the percentage of Hoechst 33258 positive cells and the caspase-3 activity in relative light units (RLUs). The right y-axis represents the OD 405 nm values from an apoptotic ELISA assay. All experiments were performed three times, each in duplicate. The data are presented as the mean ± SD. **(C)** Punctate dots of GFP indicating autophagosomes are shown by the white arrows (400x magnification).

Dominant negative RhoA-T19N, inhibitors and siRNA techniques were used to elucidate the relationships between previously identified molecules involved in ARV S1133-mediated autophagy and apoptosis. The overexpression of dominant negative RhoA and the inhibition of ROCK by Y-27632 or *ROCK1* siRNA led to a remarkable reduction in MDC positive and LC3-GFP punctuate cells: importantly, pan-caspase inhibitor, Z-VAD-FMK, did not affect the percentage of autophagic cells. Autophagy inhibitor Beclin-1 siRNA, 3-Methyladenine (3-MA), and Bafilomycin A1 also reduced the number of cells undergoing autophagy (Figure 4A).

**Figure 5:**
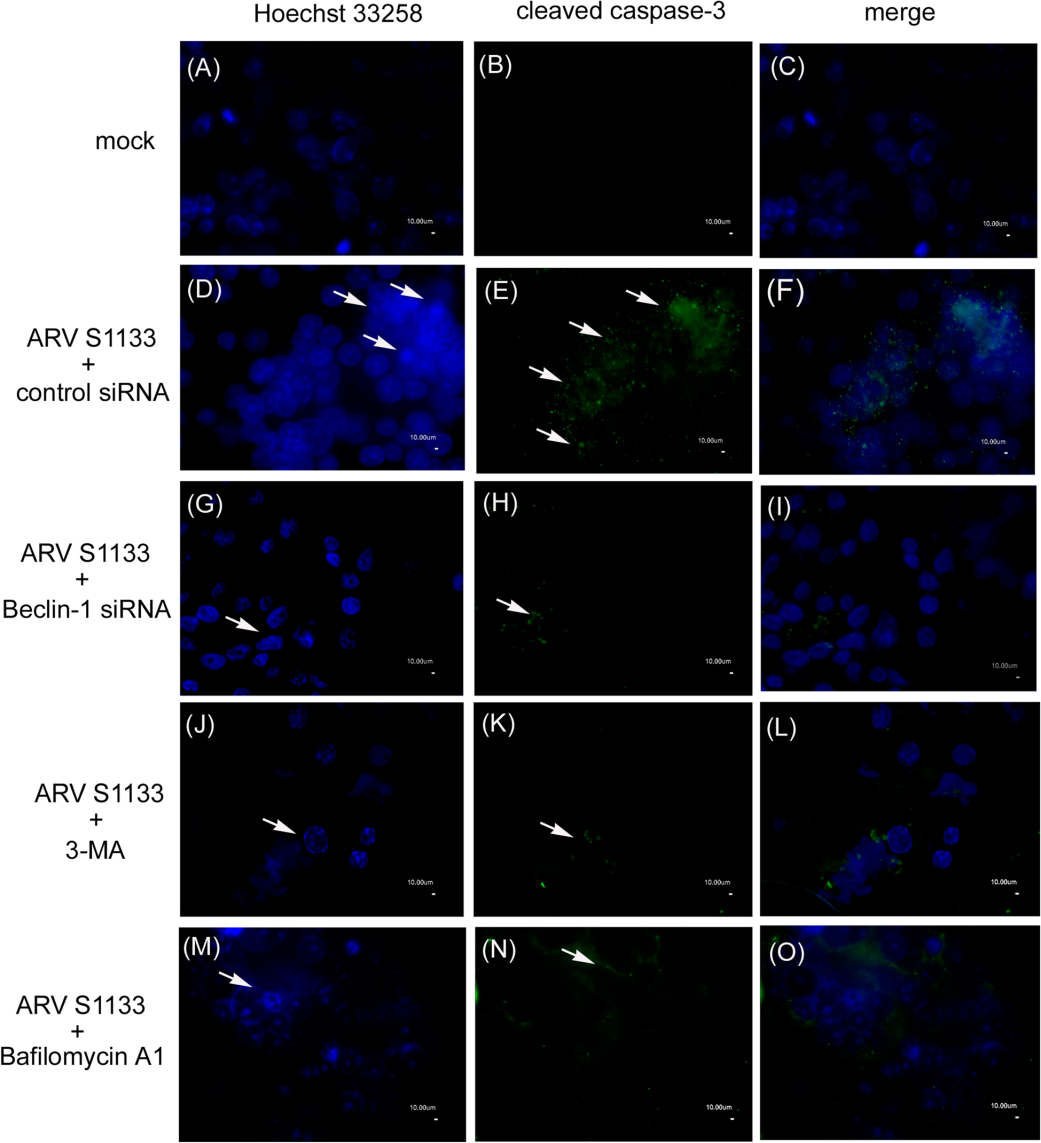
Beclin-1 is essential for ARV S1133-mediated apoptosis. Caspase-3 and Hoechst 33258 stained cells that have undergone the treatment described in Figure 4B and indicated in the left. Apoptotic cells showing condensed and fragmented DNA (A,D,G,J,M) and cleaved caspase-3(B,E,H,K,N) are indicated by arrows (400x magnification, scale bar 10 μm). Merge image of identical field upon Hoechst 33258 and cleaved caspase-3 were shown(C,F,I,L,O).

**Figure 6:**
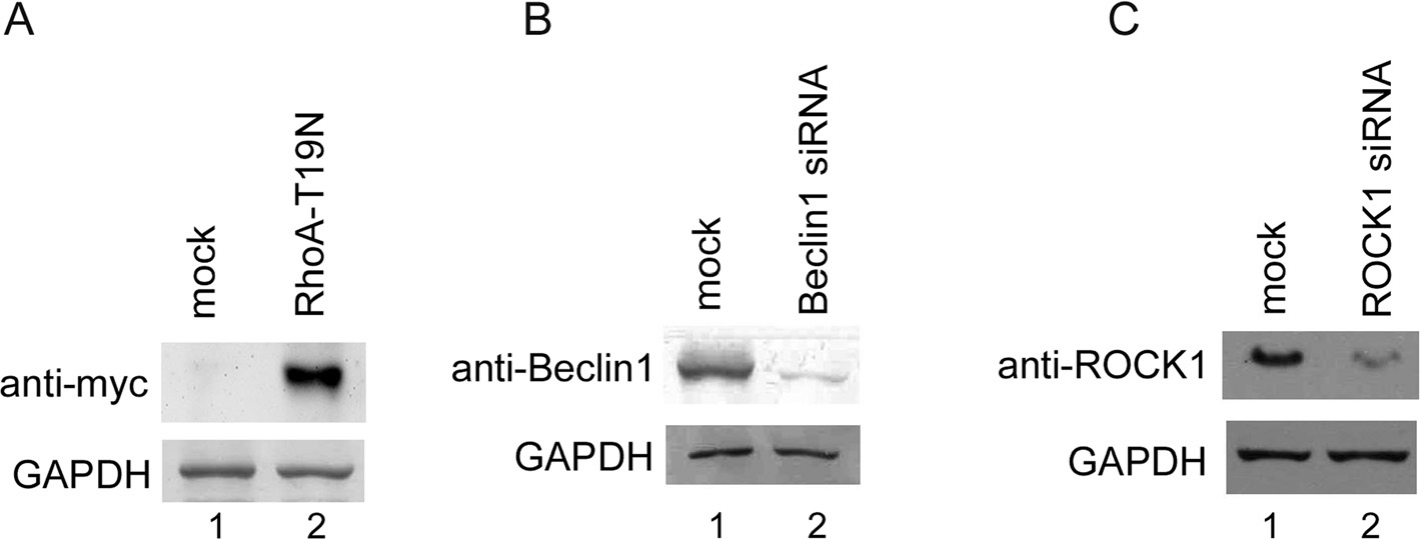
Endogenous gene knockdown efficiency confirmed by Western blotting analysis. The soluble cell lysates of Vero cells transfected with **(A)** RhoA-T19N for 48 hrs or **(B)** and **(C)** siRNA for 72 hrs were subjected to SDS-PAGE followed by antibody detection.

Next, the apoptotic characteristics of the cells were analyzed by Hoechst 33258 staining, the caspase-3 activity was assessed by chemiluminescence ELISA, and apoptotic DNA was measured by ELISA following inhibition of RhoA/ROCK1 signaling, inhibition of autophagy by *Beclin-1* siRNA, and blocking of autophagosome formation by 3-MA and Bafilomycin A1, which is a known inhibitor of the late phase of autophagy. The results of these three experiments showed that the level of apoptosis was also inhibited when the autophagic process was blocked (Figure 4B). Figure 4C shows the LC3-GFP punctate pattern of ARV S1133-infected Vero cells in the presence of various inhibitors or siRNA. The level of LC3-GFP dots significantly increased in ARV S1133-infected cells (Figure 4C, (B)), as well as in cells in the presence of caspase-3 inhibitor Z-VAD-FMK (Figure 4C, (C)). Upon inhibition of the RhoA/ROCK1 pathway, the LC3-GFP positive signals dramatically reduced (Figure 4C, (D)(E)(F)). Inhibition of autophagy mediators by *Beclin-1* siRNA, 3-MA, and Bafilomycin A1, and inhibition of the RhoA/ROCK1 pathway also significantly reduced the number of apoptotic cells, as evidenced by Hoechst 33258 staining and the immunofluorescence assay using an antibody against cleaved caspase-3 (Figure 5). The efficiency of siRNA inhibition and overexpression of dominant negative RhoA were demonstrated by Western blotting analysis (Figure 6). Taken together, these experiments clearly demonstrate that RhoA/ROCK1 signaling plays an important role in the processes of autophagy and apoptosis that occur in the early and late stages of ARV S1133 infection of cultured Vero cells.

### Functional interaction of RhoA, ROCK1 and Beclin-1 upon ARV S1133 infection

**Figure 7:**
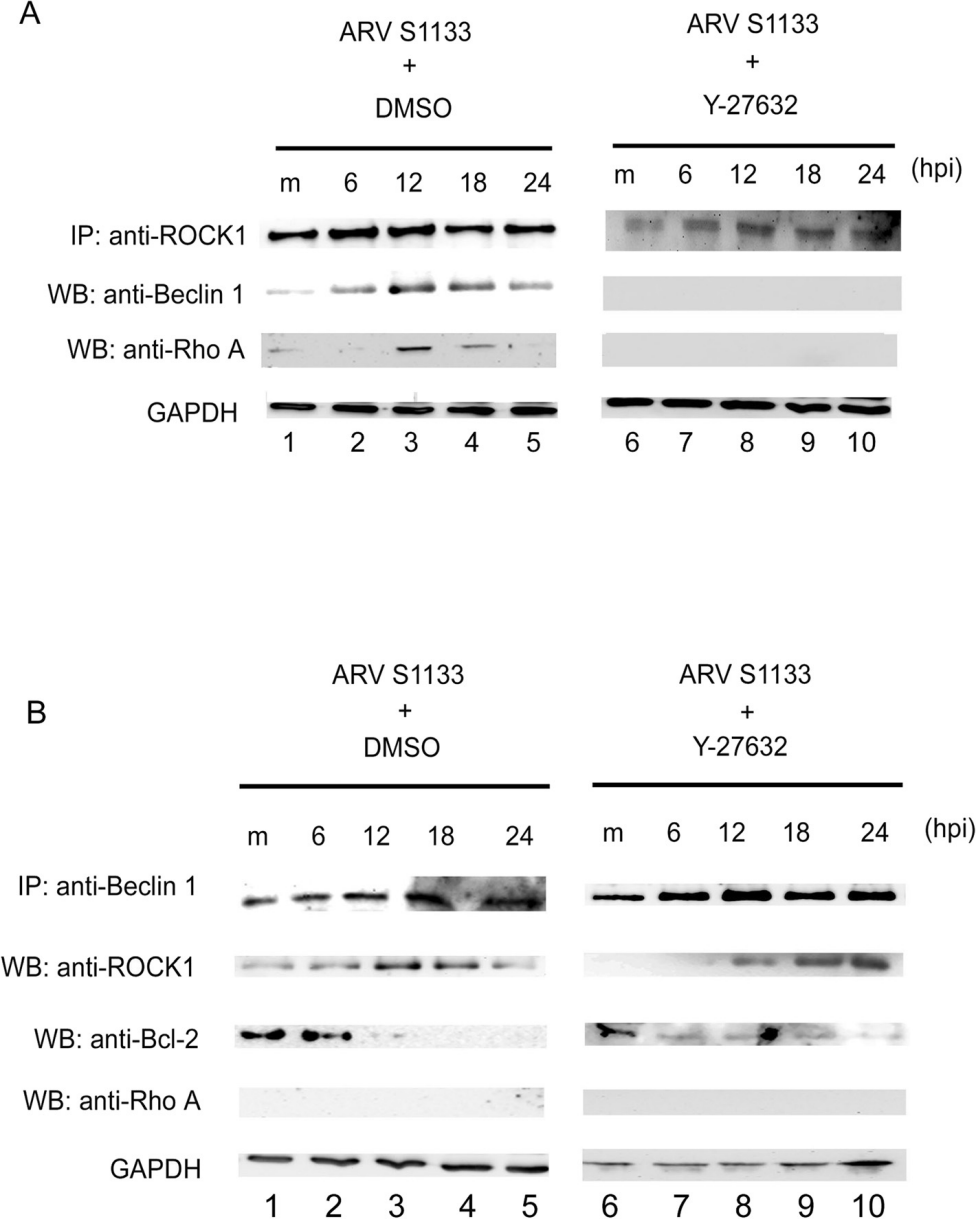
Functional interaction between RhoA, ROCK and Beclin-1 in ARV S1133-infected cells **(A, B)**. Vero cells pretreated with DMSO (left panel) or Y-27632 (right panel) for 4 hr then infected with ARV S1133 at an MOI of 5 for various incubation periods. 500 μg soluble cell lysates were precleared and immunoprecipitated with IP antibodies as indicated. The immunocomplexes were purified by protein A beads and loaded onto an SDS-PAGE gel. The interacting proteins were detected using the Western blotting technique.

To further understand the molecular mechanisms of ARV S1133-induced autophagy and apoptosis, co-immunoprecipitation followed by Western blotting analysis was utilized to detect the protein complex formation. Cell lysates from ARV S1133-infected cells with or without Y-27632 treatment were immunoprecipitated with anti-ROCK1 (Figure 7A) or anti-Beclin-1 (Figure 7B), then subjected to Western blotting analysis using the indicated various antibodies. ROCK1, Beclin-1 and RhoA formed a complex at 6–12 hpi, which corresponded to autophagy induction. In the presence of the ROCK1 inhibitor, Y-27632, the protein complex was disrupted. Thus, ROCK1 activity is essential for ARV S1133-mediated functional interactions between RhoA, ROCK1 and Beclin-1. It is known that the antiapoptotic protein, Bcl-2, interacts with Beclin-1 in non-stressed, resting cells and prevents autophagosome formation. The Bcl-2-Beclin-1 complex plays a potentially important role in the convergence of the cellular autophagic and apoptotic responses [25]. In non-stimulated Vero cells, Bcl-2 formed a complex with Beclin-1 (Figure 7B, lane 1). 6 h after ARV S1133 infection, this complex was disrupted and Beclin-1 was released, allowing it to participate in autophagy (Figure 7B, panel 3).

## Discussion

In this study, we demonstrated that ARV S1133 caused autophagy and apoptosis in Vero and DF1 cells, which was accompanied by the activation of the Beclin-1 promoter in the early to middle stages, and induction of caspase-3 expression in the middle to late stages of infection. Inhibition of autophagy by Beclin-1 knockdown or autophagy inhibitors reduced autophagy and apoptosis simultaneously. In addition, we showed that inhibition of apoptosis did not affect autophagy in ARV-infected Vero cells, and the RhoA/ROCK1 signaling pathway played an important role in the process of switching the cell from autophagy to apoptosis.

Autophagy is a dynamic process by which infected host cells clear invading viruses. A previous study by Chi and colleagues showed that inhibition of autophagy through knockdown of Beclin-1 and LC3 significantly reduced ARV replication [18]. This suggests that in the early stage of viral infection, the induction of autophagy may trigger host cells to avoid apoptotic cell death. In addition to participating in the regulation of autophagy, Beclin-1 has been demonstrated to play important roles in tumor suppression, cell death and development. Moreover, Beclin-1 also interacts with Vps34, the class III phosphatidylinositol 3-kinase (PI-3 K) that mediates autophagy and endocytosis [26]. Many studies have reported that several small GTPases are involved in, and regulate, the formation of autophagosomes [27] and apoptotic morphological changes [28]. For example, several Rab proteins have been found to be involved in different stages of autophagy [29]. In the current study, we showed the kinetics of autophagy and apoptosis in ARV S1133-infected DF1 and Vero cells, which are in agreement with those found in previous studies [17,18]. We further identified that ARV S1133 infection induces autophagy and apoptosis sequentially. ROCK1 is an effector of RhoA-GTPase and can be activated by RhoA-GTP under certain conditions [30]. A recent study by Gurkar and coworkers demonstrated that activated ROCK1 induced by stress increases the binding and phosphorylation of Beclin-1 [16]. The results of our study indicated that the expression of RhoA-GTP is dramatically increased from 12 to 30 hr post-ARV S1133 infection, and the up-regulation of phosphorylated ROCK1 also showed a similar trend. Thus, the results suggest that ARV S1133 activation of RhoA and its downstream effector ROCK1 are closely correlated with LC3 and Beclin-1 activation, as well as with cells undergoing autophagy.

We also investigated whether the autophagy process is able to regulate apoptosis in Vero cells after ARV S1133 infection. Overexpression of dominant negative RhoA or inhibition of ROCK by a specific inhibitor or siRNA, significantly reduced the number of autophagic cells; while, pre-treating cells with the pan-caspase inhibitor, Z-VAD-FMK, did not have an effect on the inhibition of autophagic activity. The expression of Beclin-1 was downregulated with *Beclin-1* siRNA, blocked autophagosome formation with 3-MA, and inhibited the late phase of autophagy with Bafilomycin A1. Inhibition of Beclin-1 dramatically reduced the level of apoptosis and the caspase-3 activity; suggesting that apoptosis was inhibited when the autophagic process was blocked (Figure 4B). This phenomenon suggests that ARV induces autophagy and apoptosis sequentially, confirming the crosstalk between autophagy and apoptosis proposed by previous researchers [31]. One of the unique aspects of our study was that multiple tools were used, from protein to cell level, to validate the autophagic and apoptotic processes. This helps to reduce bias when analyzing cell death markers after viral infection.

Beclin-1 knockdown or autophagy inhibitor 3-MA and Bafilomycin A1 treatment suppressed ARV S1133-induced autophagy and apoptosis simultaneously, suggesting a switch in cell activity from autophagy to apoptosis.

This indicates that crosstalk between autophagy and apoptosis is present in ARV S1133-infected cells. Several recent studies have also shown that crosstalk between autophagy and apoptosis may occur in cells infected with other viruses, such as the Chikungunya virus [32], enterovirus 71 [33], and ectromelia virus [34]. Although infection with different viruses may share some mechanisms, the relationship between autophagy and apoptosis is complex, as different signaling pathways are involved in these systems. For example, knockdown of autophagy genes caused enhanced apoptosis and increased viral propagation *in vitro* [32]. However, on enterovirus 71 infection, inhibition of autophagy leads to inhibit the apoptosis that is dependent on the autophagosome processing stage [33].

Our co-immunoprecipitation assay revealed that RhoA, ROCK1, and Beclin-1 formed a complex at a time point coinciding with that of autophagy induction. Taken together, these experiments clearly demonstrate that RhoA/ROCK1 signaling plays an important role in the processes of autophagy and apoptosis that occur in the early and late stages of ARV S1133-infection of Vero cells.

Previous studies have begun to elucidate the mechanisms by which cells switch from autophagy to apoptosis. One key factor is Bcl-2. Bcl-2 has been demonstrated to play a key role in the regulation of both apoptosis and autophagy, by binding to the pro-autophagic protein, Beclin-1, and pro-apoptotic proteins such as Bax [16]. The translocation of Bcl-2 from the endoplasmic reticulum to the mitochondria is an important event that regulates the switch between autophagy and apoptosis [35]. Autophagy-regulating protein 5 (ATG5) also participates in the switch from autophagy to apoptosis. In addition, to initiate autophagosome formation, ATG5 was cleaved by calpain, a calcium-dependent, non-lysosomal cysteine protease. The cleaved ATG5 fragment then translocates to mitochondria and binds to mitochondria-localized anti-apoptotic protein, Bcl-XL, and subsequently induces apoptosis [36-38].

## Conclusions

In conclusion, our results demonstrate that RhoA/ROCK1 signaling plays critical roles in the transition from autophagy to apoptosis induced by ARV S1133. The findings indicate that the important signaling molecule, RhoA, and downstream ROCK1 could be linked to the autophagy observed during ARV infection. ARV infection causes economic losses and an effectively to control the disease is required. However, the mechanisms behind ARV-induced cell death are still largely unknown. In the present study, the mechanisms involved in the ARV-mediated switch between two different types of cell death were investigated for the first time. Several critical molecules undergoing transcriptional, translational and post-translational modifications following ARV infection were identified through molecular and cellular research approaches. Whether these proposed mechanisms occur in chickens requires further study. Thus, these molecules could be considered as valuable targets for new preventive or therapeutic drugs.

## Methods

### Cell culture, virus, reagents and antibodies

The method used to culture the DF1 (a spontaneously transformed cell line of chicken embryo fibroblasts) and Vero (a normal African green monkey kidney epithelial cell line) cells followed that described in a previous study [38]. Lipofectamine 2000 (Invitrogen, Carlsbad, CA, USA) was used for RhoA-T19N plasmid transfection. ARV strain S1133 was purified using the CsCl method and quantified as previously described [39]. Anti-ROCK1 antibody was purchased from Millipore (Billerica, MA, USA). Anti-LC3, anti-cleaved caspase-3, anti-Beclin-1, and anti-GAPDH antibodies were purchased from Cell Signaling Technology Inc. (Beverly, MA, USA). Anti-p-ROCK1 (Thr455/Ser456) was obtained from Bioss (Boston, MA, USA). Y-27632, which selectively inhibits p160ROCK [40], 3-MA, Bafilomycin A1, and Z-VAD-FMK were obtained from Calbiochem (San Diego, CA, USA). Hoechst 33258 and monodansylcadaverine (MDC) were purchased from Sigma. A dominant negative RhoA-T19N plasmid with myc-tag expression was kindly provided by Dr. Jin-Mei Lai (Fu-Jen Catholic University, Taipei, Taiwan).

### Western blotting and immunoprecipitation-western blotting analysis

The Western blotting and immunoprecipitation (IP)-Western blotting procedures were carried out as described in previous studies [9,38,39,41]. Briefly, Vero cells were infected with ARV S1133 at a multiplicity of infection (MOI) of 5 for various incubation durations. Soluble total cellular lysates were collected and quantification was performed using a Bio-Rad protein assay dye. For IP-Western blotting, cell lysates were pre-cleared with Protein A beads, and then reacted with the specific antibodies. The precipitated immunocomplex was boiled, separated by SDS-PAGE electrophoresis, and transferred onto a PVDF membrane. The presence of the proteins of interest was verified by specific secondary antibodies conjugated to horseradish peroxidase (HRP). The labeled bands were then detected by adding the enhanced chemiluminescent substrate reagents (Amersham, Buckinghamshire, UK) and exposing the membrane to X-ray film.

### Luciferase assay

pGL3-Beclin-1(−644/+197, −277/+197, −58/+197)-luciferase reporter plasmids were kindly provided by Dr. Mujun Zhao (Institute of Biochemistry and Cell Biology, Shanghai, China). pRKbetaGAL containing the CMV promoter-driven β-galactosidase gene was used to normalize the transfection efficiency. A pGL3-Beclin-1-luciferase reporter plasmid and pRKbetaGAL were co-transfected into cultured Vero cells. After 24 hr of incubation, ARV S1133 at an MOI of 5 was added and the mixture incubated for an additional 24 hr. The luciferase activities were quantified using a Luciferase assay system (Promega), and the β-galactosidase activities were quantified using a β-galactosidase Enzyme Assay System (Promega). Luciferase activity was normalized to β-galactosidase activity to calculate the transfection efficiency [42].

### RhoA and ROCK activation assay

A nonradioactive Rho activation assay kit and a Rho-associated kinase (ROCK) activity assay kit were purchased from Millipore Corporation and used according to the manufacturer’s instructions. Briefly, Vero cells were infected with ARV S1133 at an MOI of 5 for different incubation durations. Cell lysates were harvested and quantified. For the Rho activation assay, lysates were added to Rho assay Reagent (Rhotekin RBD, agarose) and the reaction mixtures were incubated. Finally, Rho was analyzed by Western blotting using an anti-RhoA (clone 55) antibody and by chemiluminescent detection. ROCK inactivates myosin phosphatase through the specific phosphorylation of myosin phosphatase target subunit 1 (MYPT1) at threonine residue 696. The kit provided a plate pre-coated with recombinant MYPT1 that may be phosphorylated upon addition of an active enzyme-containing sample. A detection antibody that specifically detects only MYPT1 phosphorylated at Thr696 and an HRP-conjugated secondary detection antibody is then applied. The amount of phosphorylated substrate is measured by the addition of the chromogenic substrate, tetramethylbenzidine (TMB). The absorbance signal at 450 nm reflects the relative amount of ROCK activity in the sample.

### Hoechst 33258 and autophagic vacuoles labeled by MDC

Following the induction of autophagy by ARV S1133 infection, the cells on a coverslip were incubated with 0.1 mM MDC in phosphate-buffered saline or 1 μg/ml Hoechst 33258 at 37°C for 15 minutes [43]. After incubation, the cells were washed and mounted, and then visualized with a fluorescence microscope using an excitation filter of 360 nm and an emission filter of 525 nm for the green MDC fluorescence signal, and was an excitation wavelength of 352 nm and emission filter of 461 nm for the blue Hoechst 33285 fluorescence. MDC-positive autophagosomes revealed a punctate staining structure. The apoptotic cells showed condensed and fragmented DNA characteristics.

### siRNA techniques

ROCK1 and Beclin-1 protein knockdowns were performed using *ROCK1* siRNA and *Beclin-1* siRNA, respectively. Briefly, 5 × 10^4^ Vero cells were seeded in each well of a 6-well plate and cultured for 18 hr. The cells were then transfected with 100 nM specific or control siRNA in the presence of Oligofectamine (Invitrogen). The cells were cultured for an additional 72 hr before viral infection. *ROCK1* siRNA (M-003536-02), *Beclin-1* siRNA (M-010552-01), and non-targeting control siRNA (D-001210-01) were obtained from Dharmacon.

### Caspas-3 activity, caspase-3 staining, and apoptotic cell death ELISA

A caspase-3 activity fluorescence assay kit was purchased from Biovision and an apoptotic cell death ELISA assay kit was obtained from Roche. All procedures were conducted in accordance with the user manuals. For the caspase-3 assay, 1 × 10^6^ Vero cells were resuspended in cold cell lysis buffer before the reaction mix was added. The caspase-3 substrate, DEVD-AFC (Ac-Asp-Glu-Val-Asp-AFC; AFC = 7-Amino-4-trifluoromethylcoumarin), was added and the mixture was incubated at 37°C for 60 min. Finally, the mixture was examined by fluorescence photometry (excitation at 400 nm, emission at 505 nm) in a Modulus Multimode Reader. For the apoptotic cell death ELISA assay, cell lysates were placed in streptavidin-coated wells. The samples contained nucleosomes that reacted with anti-biotin and anti-DNA-peroxidase (POD) antibodies, then the POD activity was determined photometrically using ABTS as the substrate. Measurement of optical density (OD) was performed at 405 nm with ABTS solution as a control. Cells cultured on coverslips were fixed in 4% paraformaldehyde, permeabilized with 0.5% Triton X-100 for 5 min at room temperature, and then reacted with anti-cleaved caspase-3 antibody for 60 min at 37°C. The cells were then washed and incubated with FITC-conjugated secondary antibody. The FITC green fluorescence was visualized using an excitation filter of 360 nm and an emission filter of 525 nm.
